# *Nepenthes* chitinase *Nk*Chit2b- 1 confers broad-spectrum resistance to chitin-containing pathogens and insects in plants

**DOI:** 10.1007/s44307-025-00066-8

**Published:** 2025-04-21

**Authors:** Jun-Jie Liu, Jin-Xuan Wen, Jian-Feng Li, Feng-Zhu Wang

**Affiliations:** Guangdong Provincial Key Laboratory of Plant Stress Biology, State Key Laboratory of Biocontrol, School of Life Sciences, Sun Yat-Sen University, Guangzhou, 510275 China

**Keywords:** Chitinase, *Nk*Chit2b- 1, Pathogenic fungi, Biopesticide, Broad-spectrum resistance

## Abstract

Chitinases play critical roles in plant-pathogen/insect interactions by degrading chitin, a key structural component of fungal cell walls and insect exoskeletons. However, current research lacks comprehensive studies on the broad-spectrum disease resistance of chitinases, and novel chitinases with higher enzymatic activity remain underexplored. Here, we report the prokaryotic expression and functional characterization of *Nepenthes khasiana*-derived chitinase *Nk*Chit2b-1, demonstrating its capacity to confer broad-spectrum resistance against chitin-containing phytopathogenic fungi and insect pests. Biochemical assays revealed that *Nk*Chit2b-1 exhibits high enzymatic activity within the optimal temperature range (28–42°C) for terrestrial plant growth and the pH range (5.0–6.0) encompassing pathogen-induced apoplastic alkalization in plants. This enzymatic profile correlates with its effective inhibition of mycelial growth in major phytopathogens including *Sclerotinia sclerotiorum*, *Botrytis cinerea*, and *Magnaporthe oryzae*. Exogenous application of *Nk*Chit2b-1 conferred enhanced resistance to these pathogens in both model species (e.g., *Arabidopsis*) and crop species (e.g., tobacco, tomato, and rice). Intriguingly, *Nk*Chit2b-1 pretreatment suppressed feeding activity of brown planthopper (BPH, *Nilaparvata lugens*) nymphs on rice phloem sap and induced mortality in adult BPH upon ingestion. Furthermore, *Nk*Chit2b-1 accelerated beet armyworm (*Spodoptera exigua*) egg hatching while delaying larval development. In addition, foliar application of *Nk*Chit2b-1 on *Arabidopsis* leaves conferred antifeedant activity against beet armyworm larvae in dual-choice assays. These results collectively indicate the exceptional potential of *Nk*Chit2b-1 as an eco-friendly “green pesticide”. The exploration of novel chitinases and combinatorial chitinase strategies may overcome the limitations of single-enzyme formulations, thereby advancing chitinase applications in sustainable agriculture and plant protection.

**Supplementary Information:**

The online version contains supplementary material available at 10.1007/s44307-025-00066-8.

## Introduction

Plant diseases and insect pests constitute major constraints to global crop production, posing severe threats to food security. These biotic stressors not only reduce yield and quality of staple crops but also incur substantial economic losses worldwide, profoundly impacting sustainable development (Savary et al. 2019). Historically, disease-induced crop failures have triggered socio-economic crises, underscoring the urgent need for effective mitigation strategies (Junaid et al. 2024). Conventional pest and disease management strategies predominantly rely on chemical interventions, such as fungicides and insecticides (Strange and Scott 2005; Ristaino et al. 2021). However, the overuse of these agrochemicals has led to severe ecological consequences, including groundwater contamination, soil microbiome disruption, and unintended non-target organism toxicity, ultimately jeopardizing ecosystem stability and human health (Bardin et al. 2015). In light of these challenges, the development of sustainable and eco-friendly alternatives has become a critical priority in modern agriculture. In recent years, biological control agents have emerged as a promising solution, offering targeted and environmentally benign approaches to managing plant diseases and pests (Ntalli and Menkissoglu 2011; Woodring 2020; Xu et al. 2020; Jaiswal et al. 2022).

In agricultural production, key agricultural threats include the fungal pathogens *Botrytis cinerea*, *Sclerotinia sclerotiorum*, and *Magnaporthe oryzae*, along with insect pests beet armyworm (*Spodoptera exigua*) and brown planthopper (BPH, *Nilaparvata lugens*), posing significant threats to crop productivity. *B. cinerea* causes devastating gray mold across diverse hosts (ten Have et al. 2007), while *S. sclerotiorum* induces root rot that damages crop root systems, impairing water and nutrient uptake and ultimately stunting plant growth (Woodhall et al. 2020; Li ZX et al. 2024). *M. oryzae*, the causal agent of rice blast, can lead to extensive wilting and plant mortality, with yield reductions ranging from tens to hundreds of times under severe epidemics (Zhang et al. 2025). BPH, a phloem-feeding insect pest, directly damages rice plants by extracting sap, resulting in leaf yellowing, stunted growth, and reduced grain filling (Liu et al. 2017). Whereas beet armyworm, a globally distributed pest with over 170 host plants, compromises crop vigor by feeding on leaves and stems and facilitates viral transmission (Savary et al. 2019; Chen et al. 2019). Crucially, these pathogens and pests share chitin-containing structural components.

Chitin, a linear polymer of N-acetyl-D-glucosamine linked by β− 1,4-glycosidic bonds, is a critical structural component of fungal cell walls and arthropod exoskeletons (Zhu et al. 2016). In insects, chitin also forms the peritrophic matrix, a protective barrier in the midgut that shields against microbial invasion and mechanical damage. Disruption of this matrix by chitinases can enhance pest susceptibility to pathogens, leading to increased mortality (Reynolds and Samuels 1996; Zhang et al. 2011).

Chitinases, a class of glycoside hydrolases that catalyze the hydrolysis of β− 1,4-glycosidic bonds in chitin polymers, were first characterized nearly sixty years ago (Monreal and Reese 1969). These enzymes, along with chitinase-like proteins, are ubiquitous across life forms, from bacteria to humans (Arakane and Muthukrishnan 2010; Rathore and Gupta 2015). In plants, chitinases function as endogenous defense proteins, with their biochemical properties (e.g., pH optima) varying by origin (Hamid et al. 2013). Furthermore, these enzymes exhibit significant upregulation during plant-pathogen interactions, classifying them as pathogenesis-related (PR) proteins and underscoring their pivotal role in plant innate immunity (Zhou et al. 2019; Bartholomew et al. 2019; Bordoloi et al. 2021). By hydrolyzing chitin, the primary structural component of fungal cell walls, plant chitinases effectively suppress the growth of phytopathogenic fungi (Grover 2012). For instance, Transgenic cucumber plants harboring a rice chitinase gene *RCC2* exhibit enhanced resistance to *Botrytis cinerea*, with completely blocking appressorium-mediated penetration or restricting post-penetration colonization (Tabei et al. 1998). Co-application of a purified class IV GH19 chitinase E derived from yam tubers with commercial β− 1,3-glucanase on powdery mildew-infected strawberry fruits and leaves significantly degraded *Blumeria* pathogens and suppressed disease progression, achieving both curative and preventive effects against powdery mildew (Karasuda et al. 2003). Chitinases LbCHI32 derived from *Limonium bicolor* exhibit potent degradative activity against chitin, chitin derivatives, and the cell walls of diverse phytopathogenic fungi, including *Sclerotinia sclerotiorum*, *Fusarium oxysporum*, *Valsa sordida*, *Septoria tritici*, *Rhizoctonia solani*, and *Phytophthora sojae* (Liu et al. 2010), suggesting its potential application in enhancing plant resistance against these pathogens. Overexpression of the rice *chitinase- 3* gene in peanut (*Arachis hypogaea* L.) significantly improved resistance to the peanut brown spot pathogen *Cercospora arachidicola* (Iqbal et al. 2012). Transgenic wheat (*Triticum aestivum* L.) engineered to express the rice chitinase gene *RC24* demonstrated not only enhanced resistance to fungal stripe rust disease but also a 27–36% increase in field yield compared to wild-type wheat (Huang et al. 2013). Furthermore, transgenic expression of the bitter melon chitinase gene *McCHIT1* in rice resulted in significant resistance to sheath blight disease, with wild-type and transgenic lines exhibiting average disease indices of 92% and 37–44%, respectively. Moreover, a strong correlation was observed between McCHIT1 chitinase activity and sheath blight resistance in transgenic rice plants (Zhang et al. 2019).

The multifaceted defensive roles of chitinases extend beyond fungal suppression, with documented broad-spectrum resistance against insect and mite pests. In arthropod defense, chitinases exert resistance by targeting the β-chitin fibrillary network of insect peritrophic membranes (Reynolds and Samuels 1996; Zhang et al. 2011). Recombinant chitinase rMvEChi derived from *Myrothecium verrucaria* displayed strong anti-feeding activity and lethality against cotton bollworm (*Helicoverpa armigera*), while simultaneously inhibiting hyphal growth of the phytopathogenic fungi *Ustilago maydis* and *Bipolaris sorokiniana* (Vidhate et al. 2019). Chitinases play a pivotal role in regulating the growth and development of cotton bollworm, exhibiting stage-specific expression patterns. Disrupting the endogenous regulatory mechanisms of chitinase expression through chemical interventions significantly impaired survival rates of this pest under adverse environmental conditions or insecticide exposure (Hu et al. 2022). Notably, seed coat chitinases in soybean confer protective effects against the stored product pest *Callosobruchus maculatus*, achieving up to 90% larval mortality of *C. maculatus* when 25% purified soybean chitinase was incorporated into artificial seed coats (Silva et al. 2018). In nematode and mite management, transgenic overexpression of plant cysteine protease inhibitor CeCPI and fungal chitinase PjCHI- 1 in tomato (*Solanum lycopersicum*) conferred complete protection against all developmental stages of root-knot nematode *Meloidogyne incognita* (Chan et al. 2015). Bioactive compounds secreted from *Moringa oleifera* seeds, comprising chitinase-containing proteins, demonstrated potent inhibitory activity against soil pathogens including nematodes, achieving ovicidal activity and 100% mortality in second-stage juveniles of root-knot nematodes (Sousa et al. 2020).

Carnivorous plants, a specialized ecological group within angiosperms, have evolved as adaptations to nutrient-deficient moist or aquatic habitats. These highly specialized insectivorous plants utilize modified structures termed pitchers, organs combining physical trapping mechanisms with enzymatic digestion, to hydrolyze insect-derived chitin and access nitrogen/phosphorus nutrients, thereby circumventing habitat limitations to sustain growth (Eilenberg et al. 2006; Ellison and Lubomír 2017). The pitcher fluid, secreted by specialized glandular cells, contains a suite of hydrolytic enzymes including proteases, ribonucleases, phosphatases, and chitinases (Hatano and Hamada 2008; Ravee et al. 2021). Notably, chitinases isolated from carnivorous plants exhibit broad-spectrum activity against pathogenic and pest-derived chitins, highlighting their potential as biocontrol agents for sustainable agriculture (Renner and Specht 2012, 2013). For instance, the recombinant *Drosera rotundifolia* chitinase DrChit demonstrated significant mycelial growth inhibition rates of 40%, 43.8%, and 52.6% against *Fusarium poae*, *Trichoderma viride*, and *Alternaria solani*, respectively, while showing no significant inhibitory effects on *Rhizoctonia solani* (Rajninec et al. 2020). Furthermore, *T. viride* hyphal growth area was reduced by 43.6%–54.8% compared to wild-type controls when exposed to the crude protein extracts of transgenic tobacco overexpressing DrChit (Durechova et al. 2019). On the other hand, GH18 and GH19 family chitinases have been identified in *Nepenthes* pitcher fluids (Ishisaki et al. 2012; Renner and Specht 2012; Durechova et al. 2019), though their agronomic potential remains largely unexplored due to limited functional validation beyond enzymatic activity assays.

Significantly, chitinases pose no adverse effects on plants or mammals lacking chitin components, a target specificity that strongly supports their application in plant defense strategies. Of particular interest is chitinases derived from *Nepenthes* pitchers may exhibit potent efficiency in degrading chitin from diverse pathogens and pests, offering a potential alternative to conventional chemical pesticides.

## Materials and methods

### Plant materials and cultivation

The plants used in this study included the dicotyledonous plants *Arabidopsis thaliana,* tobacco (*Nicotiana benthamiana*) and tomato (*Solanum lycopersicum*)*,* as well as the monocotyledonous plant *Oryza sativa*. Dicotyledons, including *Arabidopsis*, were cultivated in soil (Jiffy Group, Netherlands) under controlled conditions: 65% humidity, 75 µmol m⁻^2^s⁻^1^ light intensity, 23 °C daytime/20 °C nighttime temperature cycle, and a 12-h light/12-h dark photoperiod. Rice plants were grown in an artificial climate chamber (JIUPO Biotech, China) under 200 µmol m⁻^2^s⁻^1^ light intensity, daytime temperature of 30 °C, nighttime temperature of 27 °C, and a 12-h light/12-h dark cycle.

### Microbial strains and growth conditions

*Escherichia coli *DH5α (Vazyme Biotech, China) was used for plasmid cloning, while *E. coli* BL21 (TransGen Biotech, China) served for prokaryotic expression. Both strains were cultured in LB medium supplemented with ampicillin or kanamycin at 37 °C for 12–16 h. The fungal pathogens *B. cinerea* strain 2100, *Fusarium oxysporum* strain 5176 (Fo5176) and *S. sclerotiorum* strain 1980 were cultured on potato dextrose agar (PDA) plates (Qingdao Hope Bio-Technology, China) at room temperature for 3–5 d. *M. oryzae* strain guy11 was cultured on V8 agar medium at 24 °C for 3–5 d in the dark.

### Recombinant plasmid construction

The Codon-optimized chitinase genes were synthesized (GENEWIZ, China) and cloned into an expression vector containing maltose-binding protein (MBP) and His-tag sequences via single-fragment homologous recombination (Vazyme Biotech, China). The recombinant plasmid was transformed into *E. coli* DH5α competent cells and verified by Sanger sequencing.

### Prokaryotic expression and protein purification

Plasmids from verified *E. coli* DH5α clones were extracted using a plasmid miniprep kit (Generay, China) and transformed into *E. coli* BL21 competent cells. Cultures were grown at 37 °C to an OD600 of 0.4–0.6, induced with 0.2 mM IPTG, and incubated at 16 °C for 48 h. Cells were harvested by centrifugation (8,000 rpm, 4 °C, 5 min), resuspended in NaCl buffer (20 mM Tris–HCl pH 8.0, 200 mM NaCl, 10% glycerol), and lysed via sonication (300 W, 30 min). Lysates were centrifuged (12,000 rpm, 4 °C, 20 min), and supernatants were subjected to ProteinIso® Ni–NTA resin (TransGen Biotech, China). Purified proteins were buffer-exchanged into storage buffer (20 mM Tris–HCl pH 8.0, 10% glycerol) using the Aminon Ultra- 15 30 K Centrifugal Filter Device (Merck Millipore, USA). The storage buffer was also used as “Mock buffer” in the following experiments.

### Protein quantification and enzymatic activity assays

Protein concentration was determined using the bicinchoninic acid (BCA) assay (TransGen Biotech, China). Chitinase activity was measured using a chitinase assay kit (Solarbio science technology, China) by incubating 50 µM chitinase under varying pH (3.0, 4.0, 5.0, 5.6, 6.0, 7.0, and 8.0) and temperature (4 °C, 16 °C, 24 °C, 28 °C, 37 °C, 42 °C, 50 °C) conditions and measuring residual activity. The pH of the reaction mixture was adjusted using a series of buffers: 0.04 M citric acid-sodium citrate (pH 3.0–5.6), 0.04 M Na_2_HPO_4_-NaH_2_PO_4_ (pH 6.0–8.0). One unit of activity was defined as the amount of enzyme producing 1 μmol N-acetylglucosamine (NAG) per hour.

### Antifungal assays

A 20 µL aliquot of 5 µM *Nk*Chit2b- 1 or Mock buffer was applied to medium, and 5 mm fungal plugs were inverted on the droplets. Mycelial growth was monitored and photographed daily. Mycelial areas were quantified using ImageJ software. Biological replicates (n ≥ 3) were performed for each treatment.

### Pathogen inoculation

#### S. sclerotiorum

5 mm Mycelial plugs from actively growing PDA cultures were inoculated onto *Arabidopsis*, tobacco, or tomato leaves pre-treated with 5 µL of 8 µM *Nk*Chit2b- 1 or Mock buffer. Leaves were maintained on 0.8% plant agar plates in darkness for 36 h.

#### B. cinerea

Spore suspensions (1 × 10^6^ CFU/mL) were incubated with 8 µM NkChit2b- 1 or mock buffer and applied to *Arabidopsis* leaves on 0.8% agar plates. Plates were kept in darkness for 36 h.

#### M. oryzae

A spore suspension of *M. oryzae* (1 × 10^6^ CFU/mL) was prepared. Middle segments of healthy 4-week-old rice leaves were gently wounded using a pipette tip. A 5 µL aliquot of spore suspension containing 5 µM *Nk*Chit2b- 1, 10 µM *Nk*Chit2b- 1, or Mock buffer was applied to each wound. Each leaf was inoculated at seven points. The inoculated leaves were incubated in the dark at 28 °C for 2 days, followed by a 12-h light/12-h dark cycle for 2–3 days before imaging. Disease areas were measured using ImageJ.

### *Nk*Chit2b- 1 treatment of BPH

Eighteen third-instar BPH nymphs were weighed and starved for 2 h. The nymphs were then placed in pre-weighed sealed bags connected to rice leaf sheaths pre-treated with 5 µM *Nk*Chit2b- 1 or Mock buffer. After 24 h, the bags and nymphs were re-weighed, and the difference in weight was recorded as honeydew production (Shen et al. 2022). For survival assays, BPH nymphs and adults were placed in glass tubes with one side darkened and the other side illuminated, containing 5% sucrose solution supplemented with 5 µM NkChit2b- 1 or Mock buffer. Survival rates were recorded after 24 h.

### *Nk*Chit2b- 1 treatment of beet armyworm

Beet armyworm eggs were sprayed with 5 µM *Nk*Chit2b- 1 or Mock buffer, and hatching rates were assessed after 24 h. Larvae were fed artificial diet supplemented with 5 µM *Nk*Chit2b- 1 or Mock buffer, with growth parameters monitored daily. For behavioral assays, 20 first-instar larvae were placed at the center of 15 cm Petri dishes containing 0.8% agar, with alternating 5 µM NkChit2b- 1-treated and mock-treated *Arabidopsis* leaf halves arranged peripherally. Larval distribution on each leaf type was recorded after 24 h.

## Results

### *Nk*Chit2b- 1 exhibits high chitinase activity under plant physiological conditions

**Fig. 1:**
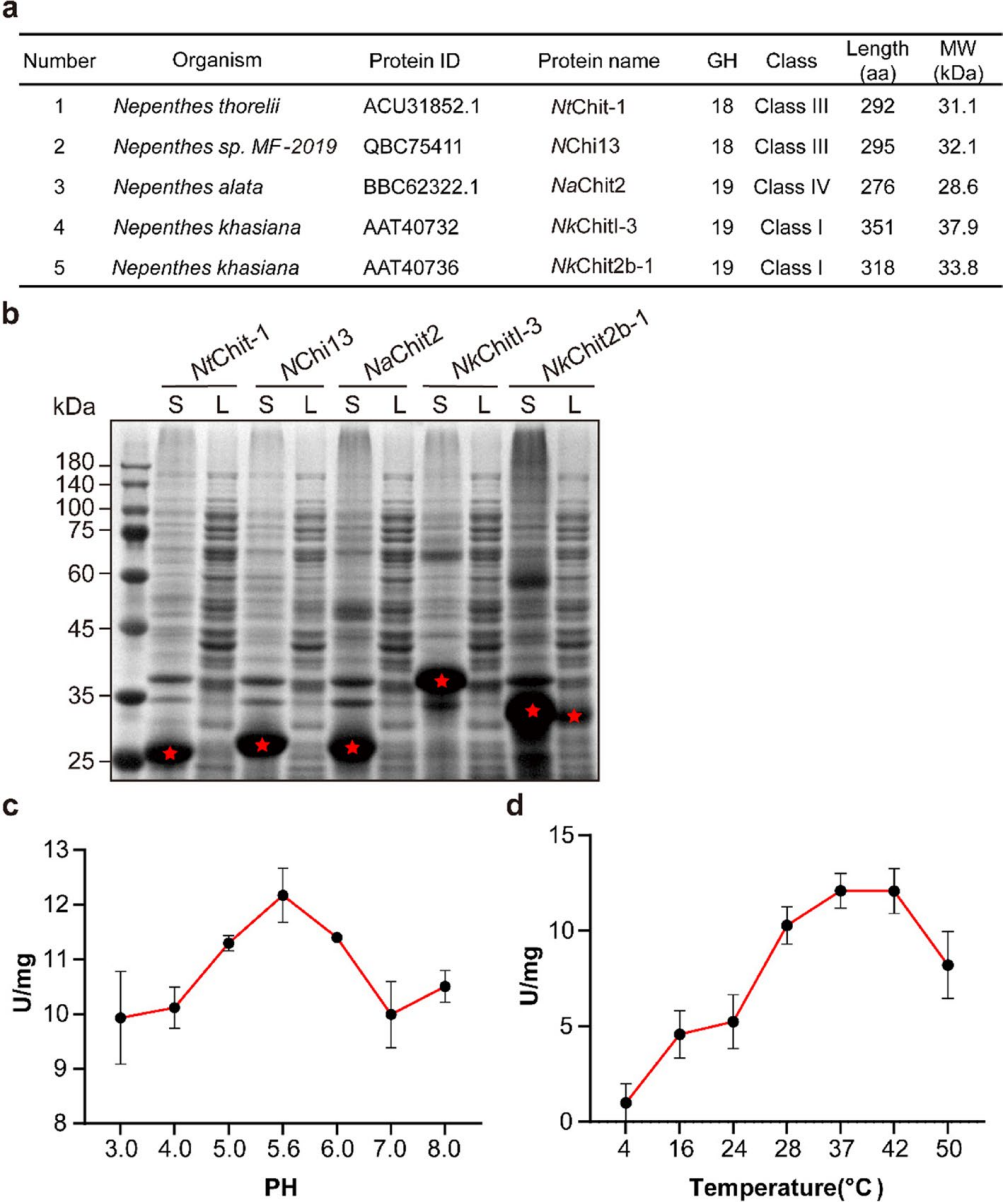
Enzymatic activity profiling of chitinase *Nk*Chit2b- 1 from *Nepenthes. ***a** Predicted information of five *Nepenthes* chitinases from the UniProt database. GH, glycosyl hydrolase. **b** Heterologous expression of five *Nepenthes* chitinases in a prokaryotic system. M, marker; S, sediment; L, liquid supernatant. Asterisks denote target proteins. **c** Effect of pH on the enzymatic activity of *Nk*Chit2b- 1. Activity was assayed at pH 3.0, 4.0, 5.0, 5.6, 6.0, 7.0, and 8.0. **d** Effect of temperature on the enzymatic activity of *Nk*Chit2b- 1. Activity was evaluated at 4 °C, 16 °C, 24 °C, 28 °C, 37 °C, 42 °C, and 50 °C. Data are presented as mean ± SEM (*n* = 3)

Plant chitinases are phylogenetically categorized into seven classes based on amino acid sequence variations. According to classification criteria, classes I, II, IV, VI, and VII belong to the GH19 glycosyl hydrolase family, whereas classes III and V are categorized under the GH18 family (Roopavathi et al. 2015). Previous studies have identified five chitinases from *Nepenthes* plants, including three GH19 family members (*Nk*Chit2b- 1, *Nk*Chitl- 3, and *Na*Chit2) and two GH18 family enzymes (NChi3 and acidic endo-chitinase), as shown in Fig. 1a (Rottloff et al. 2011; Renner et al. 2012). To identify candidate chitinases with biocontrol potential, we intend to perform functional characterization of these five chitinases. However, subsequent heterologous expression in *E. coli* revealed that only the 33.8-kDa *Nk*Chit2b- 1 from *Nepenthes khasiana* was successfully purified in soluble form, while the others formed insoluble inclusion bodies (Fig. 1b). Biochemical analysis demonstrated that *Nk*Chit2b- 1 exhibited optimal chitinase activity within the pH range of 5.0–6.0 (Fig. 1c), which aligns with the extracellular pH values of plant cells under both basal conditions and pathogen-induced alkalization (Masachis et al. 2016). Moreover, the enzyme maintained high activity across a temperature range of 28–42 °C (Fig. 1d), corresponding to the typical mesophilic environments encountered by plants. These findings collectively suggest that *Nk*Chit2b- 1 possesses exceptional catalytic efficiency within the pH and thermal regimes encountered during plant growth and pathogen defense responses, positioning it as a promising candidate for the development of biopesticide formulations. In parallel, we assayed the enzymatic activities of two additional plant chitinases exhibiting homology to *Nk*Chit2b- 1, specifically *At*HCHIB from *Arabidopsis thaliana* and *Os*Chit1 from *Oryza sativa* (Samac et al. 1990; Xu et al. 1996). Unexpectedly, both chitinases displayed comparable enzymatic activity to *Nk*Chit2b- 1 across tested pH values (3.0–8.0) and temperature ranges (4–50 °C) (Fig. S1a, 1b). Nevertheless, when compared prokaryotic expression yields, *Nk*Chit2b- 1 achieved over ten-fold higher recombinant protein production efficiency than the other two chitinases (Fig. S1c). Considering both production costs and application potential, *Nk*Chit2b- 1 was selected for further characterization.

### *Nk*Chit2b- 1 confers significant resistance to *S. sclerotiorum* in *Arabidopsis* and Tomato

**Fig. 2:**
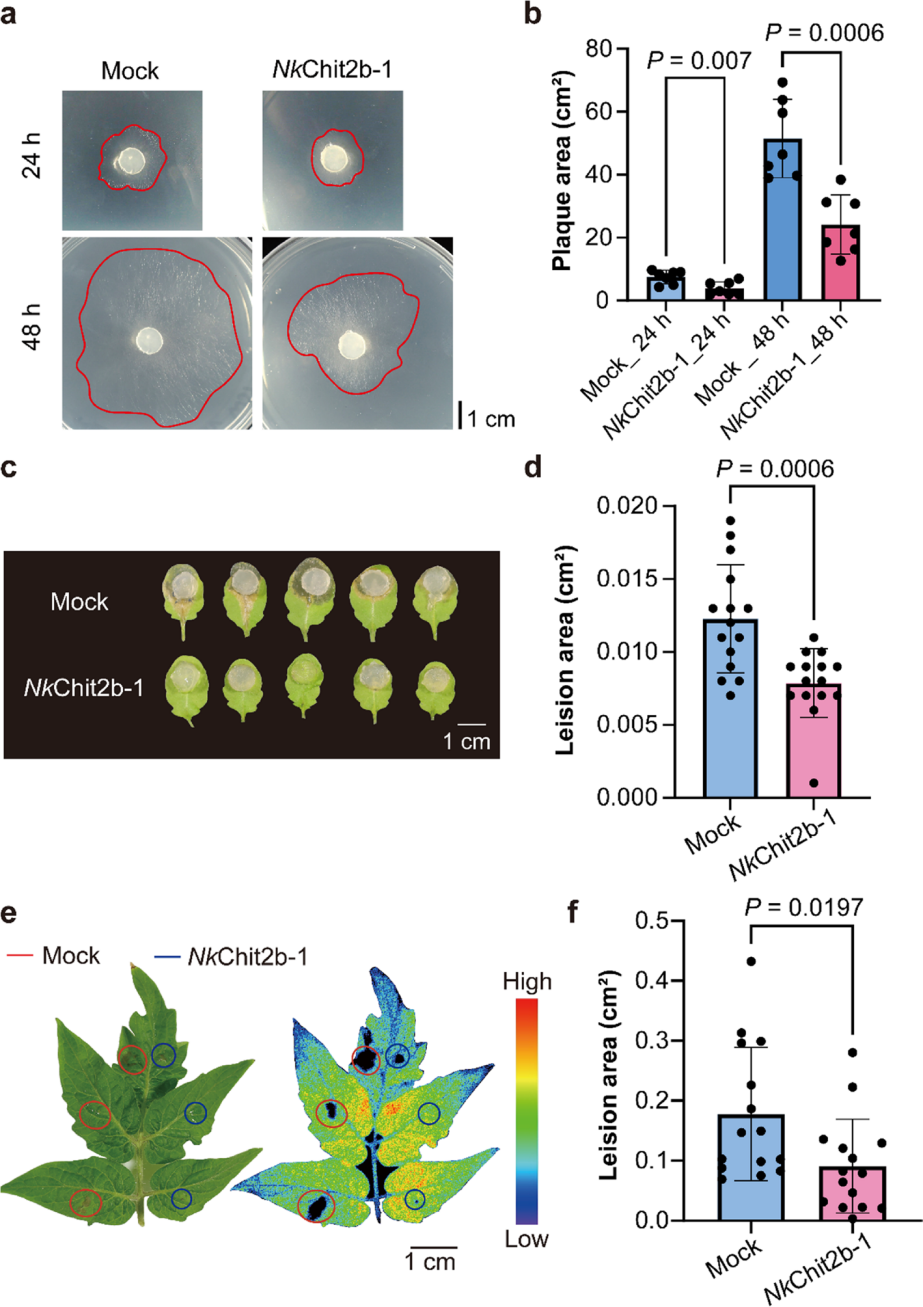
Antifungal activity of *Nk*Chit2b- 1 against *S. sclerotiorum* and its role in disease resistance. **a ***Nk*Chit2b- 1 effectively inhibits mycelial growth of *S. sclerotiorum.* Representative images show mycelial growth at 24 h and 48 h post-inoculation. Scale bar = 1 cm. **b** Quantitative analysis of the *S. sclerotiorum* plaque areas. Data are presented as mean ± SEM (*n* = 7). **c ***Nk*Chit2b- 1 enhances resistance to *S. sclerotiorum* in *Arabidopsis*. Disease symptoms in *Arabidopsis* leaves were observed 24 h after inoculation. Scale bar = 1 cm. **d** Statistical analysis of disease lesion areas in *Arabidopsis*. Data are presented as mean ± SEM (*n* = 15). **e ***Nk*Chit2b- 1 enhances tomato leaf resistance to *S. sclerotiorum*. Representative images show tomato leaves 48 h after inoculation. The right image shows in vivo imaging of cell death. Scale bar = 1 cm. **f** Statistical analysis of disease lesion areas in tomato. Data are presented as mean ± SEM (*n* = 15). Statistical significance was assessed using a two-tailed Student’s unpaired t-test, with *P* indicating the significance of differences

*S. sclerotiorum*, an *Ascomycota* plant pathogen, infects over 400 plant species across *Brassicaceae*, *Fabaceae*, *Solanaceae*, *Asteraceae*, and *Apiaceae* families, posing a severe threat to global agriculture (Woodhall et al. 2020). Previous studies have reported the antifungal activity of plant chitinases (Tabei et al. 1998; Karasuda et al. 2003; Liu et al. 2010; Zhang et al. 2013; Kabir et al. 2016). In this study, we investigated the functional role of *Nk*Chit2b- 1 in mediating plant resistance to *S. sclerotiorum*. Time-course analysis of mycelial growth inhibition revealed that *Nk*Chit2b- 1 significantly suppressed *S. sclerotiorum* hyphal expansion in vitro, with enhanced efficacy observed over time (Fig. 2a, b).

To evaluate its potential as a biocontrol agent, we examined whether exogenous application of *Nk*Chit2b- 1 could enhance plant resistance. In *Arabidopsis*, co-treatment with *Nk*Chit2b- 1 and *S. sclerotiorum* resulted in significantly smaller necrotic lesion areas compared to untreated controls (Fig. 2c, d), indicating that *Nk*Chit2b- 1 enhances resistance to *S. sclerotiorum*. Tomato, one of the top three globally traded vegetables, is severely affected by *S. sclerotiorum*-induced stem rot, a major disease in tomato cultivation worldwide (Farzand et al. 2019). Similarly, *Nk*Chit2b- 1 treatment conferred enhanced resistance to *S. sclerotiorum* in MicroTom tomato variety, as evidenced by reduced lesion severity (Fig. 2e, f). These results establish that *Nk*Chit2b- 1 not only exerts potent antifungal activity against *S. sclerotiorum* mycelium but also demonstrates broad-spectrum protective effects in agriculturally important host plants, suggesting its potential as a universal biocontrol agent against sclerotinial pathogens.

### *Nk*Chit2b- 1 confers significant resistance to *B. cinerea* in plants

**Fig. 3:**
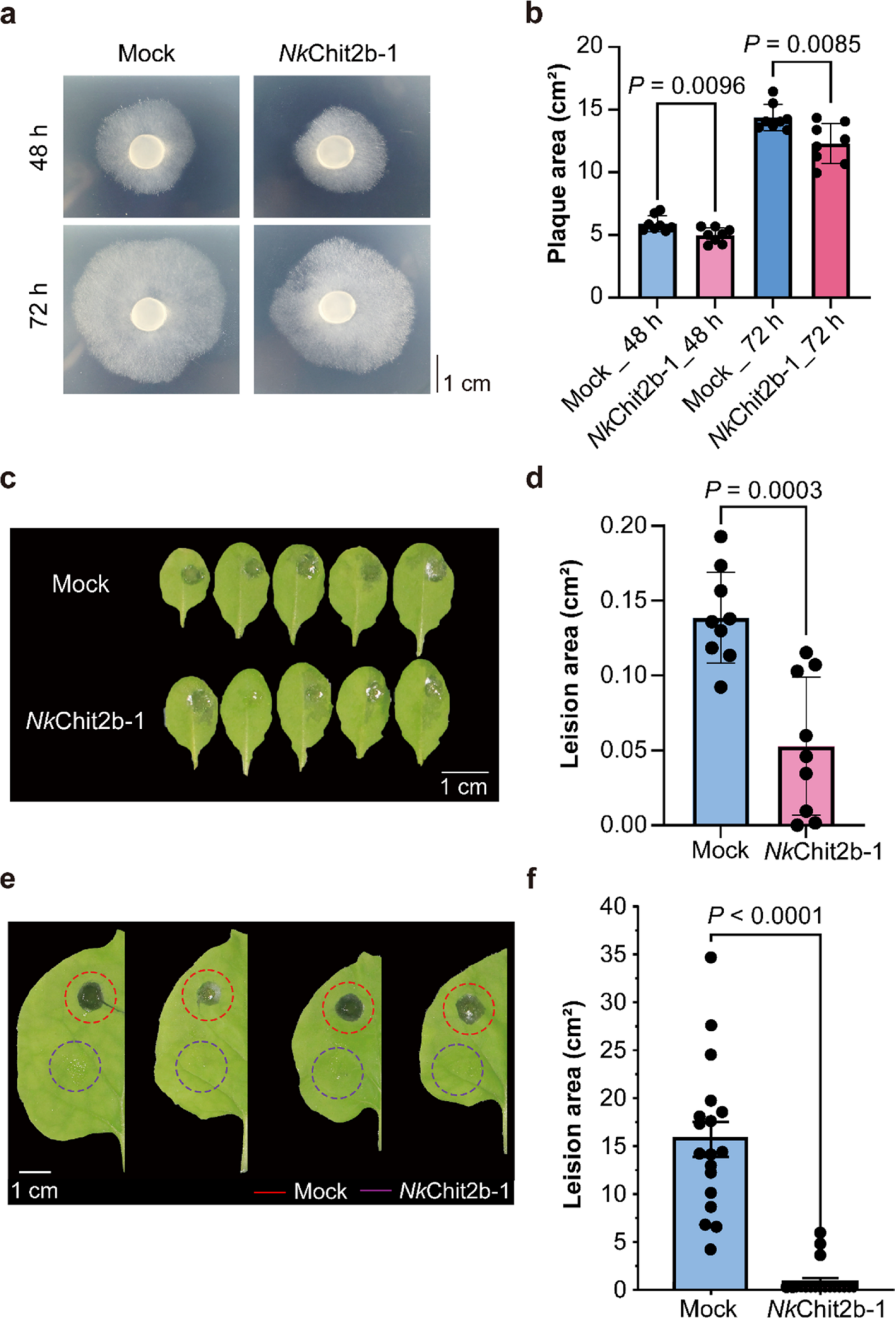
Antifungal activity of *Nk*Chit2b- 1 against *B. cinerea* and its role in disease resistance. **a ***Nk*Chit2b- 1 effectively inhibits the mycelial growth of *B. cinerea*. Representative images show mycelial growth at 48 h and 72 h prost-inoculation. Scale bar = 1 cm. **b** Quantitative analysis of *B. cinerea* plaque area. Data are presented as mean ± SEM (*n* = 8). **c** 8 μM *Nk*Chit2b- 1 enhances resistance to *B. cinerea* in *Arabidopsis*. Disease symptoms in *Arabidopsis* leaves were observed 24 h after inoculation. Scale bar = 1 cm. **d** Statistical analysis of disease lesion areas in *Arabidopsis*. Data are presented as mean ± SEM (*n* = 15). **e** 3 μM *Nk*Chit2b- 1 enhances resistance to *B. cinerea* in tobacco. Representative image show tobacco leaves 24 h after inoculation. scale bar = 1 cm. **f** Statistical analysis of disease lesion areas in tobacco. Data are presented as mean ± SEM (*n* = 15). Statistical significance was assessed using a two-tailed Student’s unpaired t-test, with *P* indicating the significance of differences

*B. cinerea*, a filamentous fungus causing devastating diseases in diverse crops including tomato, grape, and strawberry, leads to severe yield losses worldwide (ten Have et al. 2007). To evaluate the broad-spectrum antifungal potential of *Nk*Chit2b- 1, we evaluated its inhibitory effects on *B. cinerea*. Co-inoculation assays revealed that *Nk*Chit2b- 1 significantly suppressed *B. cinerea* mycelial growth in vitro, with the enhanced inhibitory efficacy observed over time (Fig. 3a, b). Co-inoculation of *Arabidopsis* leaves with *B. cinerea* and 8 μM *Nk*Chit2b- 1 reduced leaf lesion area by 61.9% compared to controls inoculated with *B. cinerea* alone (Fig. 3c, d). Treatments combining *B. cinerea* with 2 μM or 10 μM *Nk*Chit2b- 1 exhibited reductions of 25.8% and 95.6% in lesion area, respectively (Fig. S2). These results demonstrate that *Nk*Chit2b- 1 confers dose-dependent protection against *B. cinerea *infection in *Arabidopsis.* Similarly, when tobacco was co-inoculated with *Nk*Chit2b- 1 and *B. cinerea*, a marked attenuation of disease symptoms was observed (Fig. 3e, f). These results demonstrate that *Nk*Chit2b- 1 not only exerts time-dependent mycelial growth inhibition of *B. cinerea* but also confers cross-plant resistance in both *Arabidopsis* and tobacco, highlighting its promising role as a universal antifungal agent for crop protection.

### *Nk*Chit2b- 1 confers significant resistance to rice blast pathogen

**Fig. 4:**
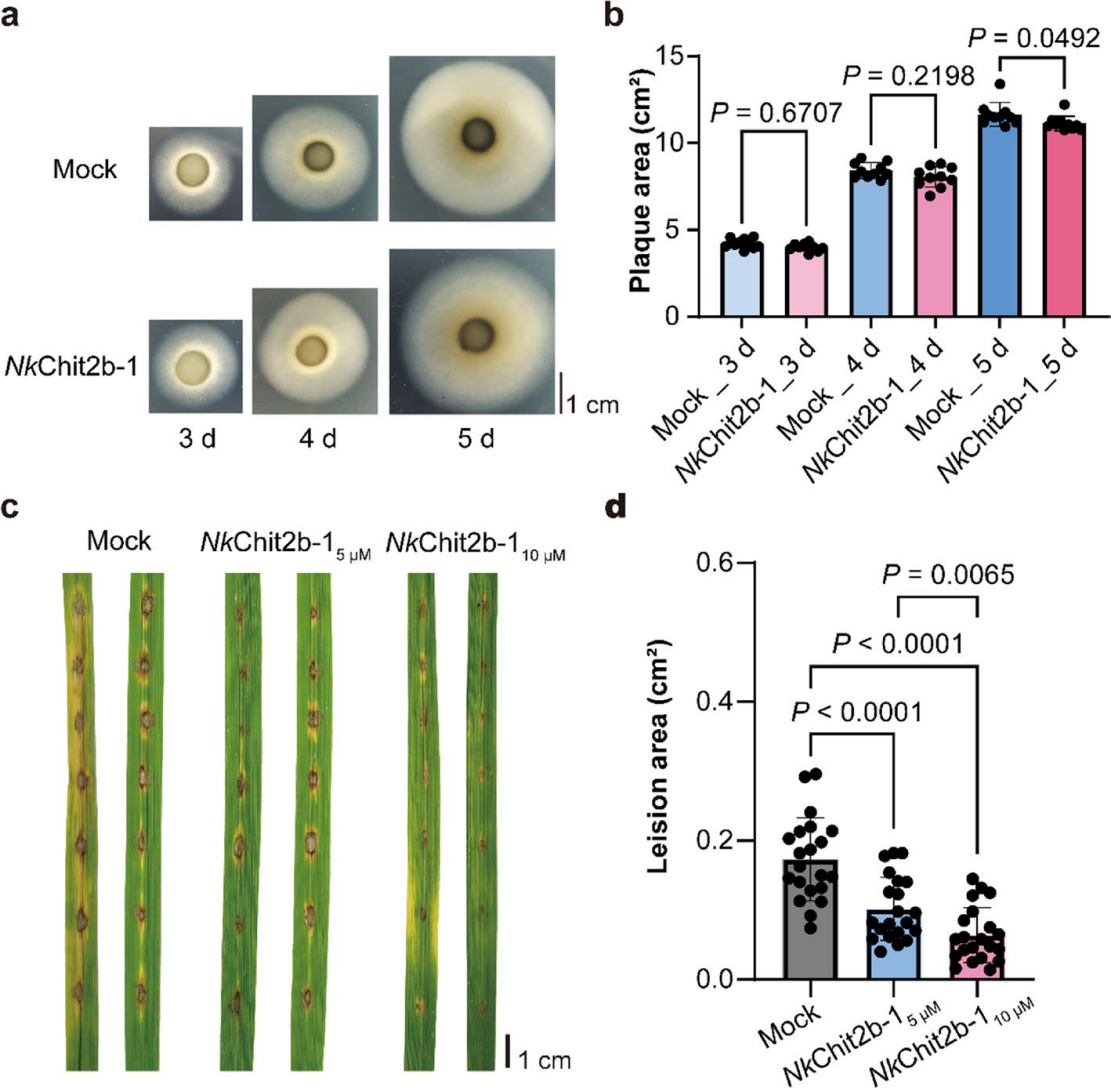
Inhibitory effects of *Nk*Chit2b- 1 on rice blast caused by *M. oryzae.* **a** *Nk*Chit2b- 1 inhibits mycelial growth of *M. oryzae*. Representative images show mycelial growth at 3 d, 4 d, and 5 d post-inoculation. Scale bar = 1 cm. **b** Quantitative analysis of *M. oryzae* plaque area. Data are presented as mean ± SEM (*n* = 9). **c** *Nk*Chit2b- 1 enhances resistance to *M. oryzae* in rice. Representative image shows rice leaves 5 days after inoculation. Scale bar = 1 cm. **d** Statistical analysis of disease lesion areas in rice. Data are presented as mean ± SEM (*n* = 21). **b, d** Data were processed using one-way ANOVA, with *P* indicating the significance of differences

Previous studies demonstrated that *Nk*Chit2b- 1 exerts broad-spectrum antifungal activity against pathogenic fungi in dicotyledonous plants. To investigate its potential against monocotyledonous pathogens, we evaluated the inhibitory effects of *Nk*Chit2b- 1 on *M. oryzae*, which causes rice blast, one of the three most devastating rice diseases globally. Co-inoculation assays revealed that *Nk*Chit2b- 1 significantly suppressed *M. oryzae* mycelial growth in vitro, with enhanced inhibition observed over time (Fig. 4a, b). Notably, at 5 days post-inoculation, treated hyphae exhibited notably sparse marginal growth patterns compared to controls (Fig. 4a, b).

Co-treatment of rice plants (*Oryza sativa* cv. Zhonghua 11, ZH11) with *Nk*Chit2b- 1 and *M. oryzae* significantly reduced leaf lesion areas compared to the controls (Fig. 4c, d). Moreover, a dose-dependent relationship was observed, where higher concentrations of *Nk*Chit2b- 1 conferred greater resistance to rice blast (Fig. 4c, d). These results demonstrate that *Nk*Chit2b- 1 not only inhibits *M. oryzae* mycelial growth through time-dependent mechanisms but also enhances rice blast resistance in a concentration-dependent manner, suggesting its potential as a universal antifungal agent for cereal crops.

### *Nk*Chit2b- 1 reduces BPH infestation in rice

**Fig. 5:**
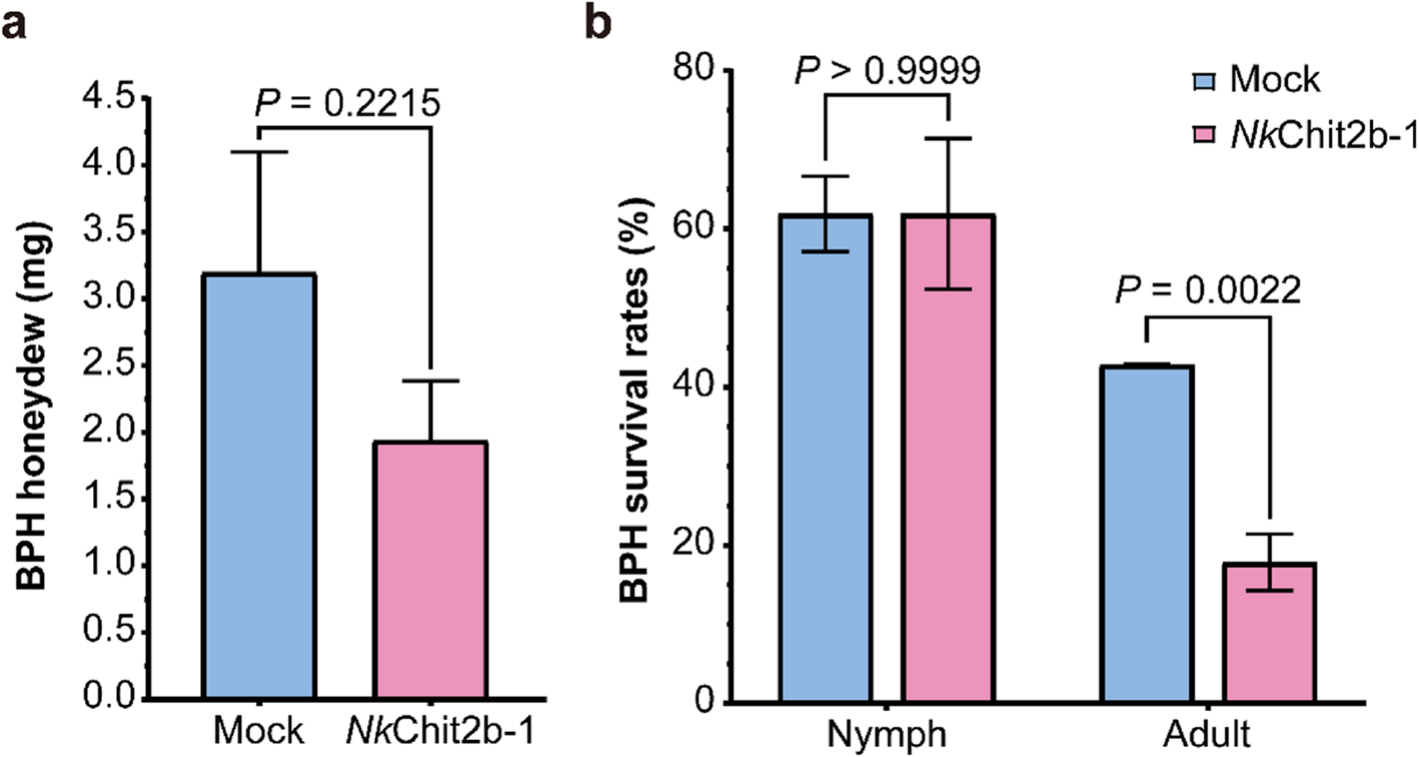
Effects of *Nk*Chit2b- 1 on BPH. **a ***Nk*Chit2b- 1 consumption reduces honeydew production in BPH. Data are presented as mean ± SEM (*n* = 18). **b** Survival rates of nymphs and adults. Data are presented as mean ± SEM (*n* = 18). Statistical significance was assessed using a two-tailed Student’s unpaired t-test, with *P* indicating the significance of differences

Rice production faces dual threats from fungal pathogens and insect pests. The BPH, a monophagous pest specific to rice, causes catastrophic yield losses (Liu et al. 2017). This insect feeds via stylet penetration into plant vascular tissues, absorbing nutrients including water, sugars, and minerals. Due to incomplete absorption, excess sugars, such as glucose and fructose, are excreted as honeydew through anal pores (Zhu et al. 2020). To assess the impact of *Nk*Chit2b- 1 on BPH feeding, we placed third-instar nymphs on rice sheaths pretreated with *Nk*Chit2b- 1 and collected honeydew after 24 h. We found that nymphs feeding on *Nk*Chit2b- 1-treated sheaths produced less honeydew compared to those on control sheaths (Fig. 5a), indicating impaired nutrient uptake and/or metabolic interference.

To further investigate whether *Nk*Chit2b- 1 affects the peritrophic matrix (PM) of BPH, we provided both nymphs and adults with sucrose solution containing *Nk*Chit2b- 1 under light-induced conditions. After 24 h, we observed minimal mortality in nymphs but significant mortality in adults (Fig. 5b). This differential response may relate to structural and functional differences in the PMs of life stages. Nymphal PMs, characterized by thinner and more flexible chitin layers, enable efficient nutrient absorption during short-term feeding (Wang et al. 2004; Kelkenberg et al. 2015). In contrast, adult PMs possess thicker chitin matrices that provide mechanical protection during prolonged feeding (Nakashima et al. 2018). Thus, the disruption of the PM by *Nk*Chit2b- 1 has a more pronounced impact on adult survival. Since only adult BPH are capable of long-distance migration, the use of *Nk*Chit2b- 1 could further reduce the spread of BPH, safeguarding rice production and food security.

### *Nk*Chit2b- 1 alleviates damage caused by beet armyworm on plants

**Fig. 6:**
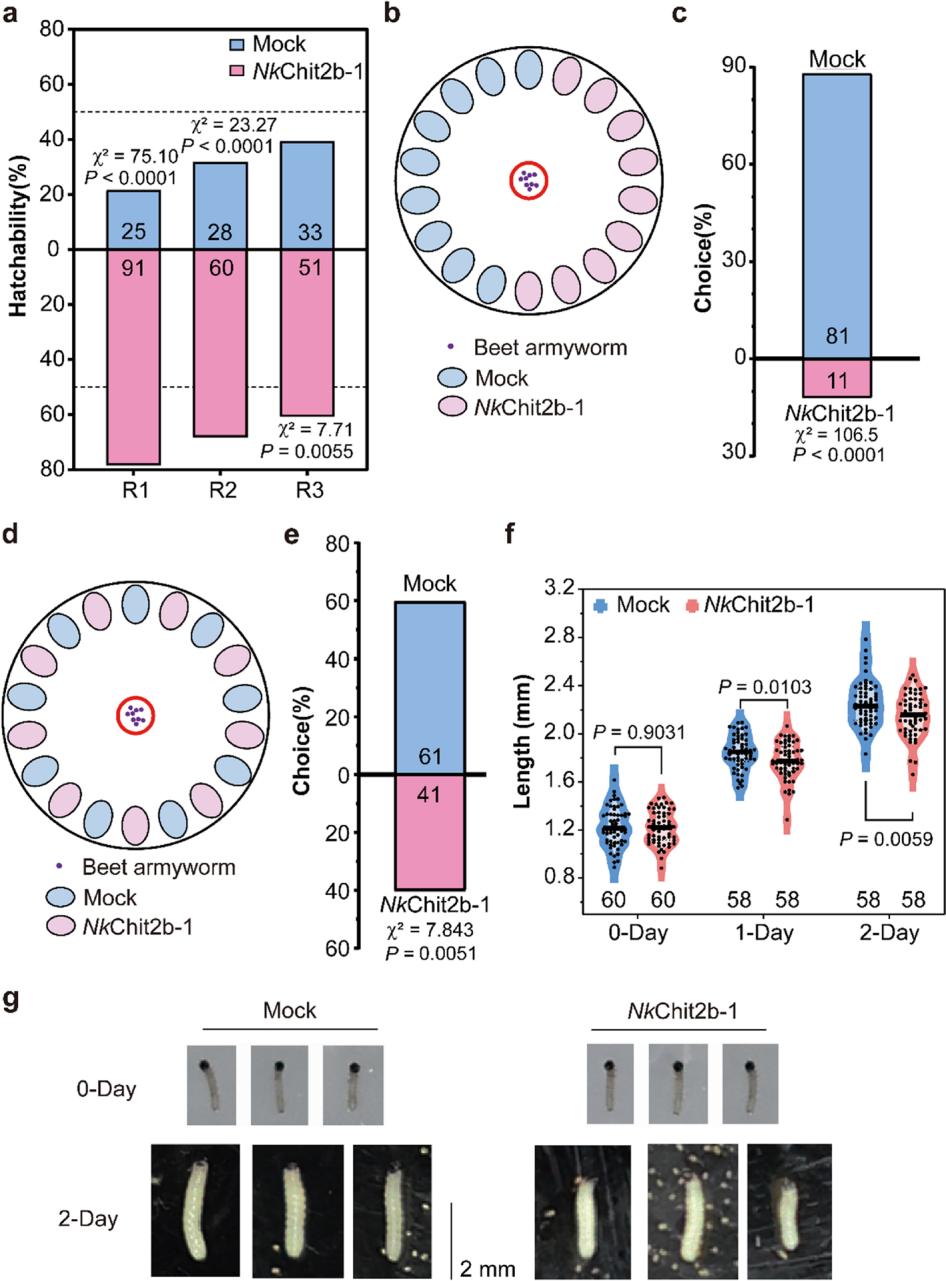
Effects of *Nk*Chit2b- 1 on the growth and feeding behavior of beet armyworm**. a ***Nk*Chit2b- 1 disrupts the hatching cycle of beet armyworm. Hatching rates were quantified across three biological repeats (*n* = 20). Data are presented as mean ± SEM. *Nk*Chit2b- 1 affects feeding behavior of beet armyworm. **b**,** d** Leaf arrangements under different treatments. **c**, **e** Statistical analysis of feeding rates. Data are presented as mean ± SEM (*n* = 20). *Nk*Chit2b- 1 influences development of beet armyworm. **f** Statistical analysis of the larval length of beet armyworm. Larvae fed with 5 µM *Nk*Chit2b- 1 or Mock solution were measured at 0 d, 1 d, and 2 days. Data are presented as mean ± SEM (*n* = 58 or 60). Statistical significance was assessed using a two-tailed Student’s unpaired t-test. **g** Larval growth status. Representative images of beet armyworm larvae fed with *Nk*Chit2b- 1 and Mock at 0 and 2 days. Scale bar = 2 cm. Data were statistically analyzed using the χ2 tests. χ2 reflects the difference between observed and expected values. The larger the value, the more significant the difference. The *P* values indicate the significance of differences

The beet armyworm is a polyphagous agricultural pest that inflicts significant damage on economically important crops such as sugar beet, cotton, maize, tobacco, and various vegetables (Greenberg et al. 2001). Its multifaceted damage includes foliar feeding, growth inhibition, fruit impairment, and pathogen transmission, posing a serious threat to agricultural production (Saeed et al. 2010). We first investigated the impact of *Nk*Chit2b- 1 on beet armyworm egg hatching. Results showed that *Nk*Chit2b- 1 accelerated the hatching process of beet armyworm eggs (Fig. 6a). The eggshell, composed of cross-linked chitin, typically protects the embryo from environmental stressors such as desiccation and temperature fluctuations (Kaliappanadar et al. 2022). Treatment with *Nk*Chit2b- 1 likely disrupted the chitinous eggshell, leading to premature hatching and abnormal developmental cycles, which may negatively affect larval development. Additionally, accelerated egg hatching in beet armyworm may destabilize pest population dynamics, as premature larvae face higher environmental mortality.

Subsequently, we investigated the impact of *Nk*Chit2b- 1 on larval feeding behavior. When given a choice between *Nk*Chit2b- 1-treated and untreated *Arabidopsis* leaves arranged alternately or split evenly on a circular plate, beet armyworm larvae significantly preferred untreated leaves (Fig. 6b-e). This suggests that exogenous application of *Nk*Chit2b- 1 alters larval feeding preferences, potentially due to its effects on larval growth and development. To validate this hypothesis, we assessed the long-term effects of *Nk*Chit2b- 1 on larval growth. Larvae fed an artificial diet containing *Nk*Chit2b- 1 exhibited delayed development, with the effect becoming more pronounced over time (Fig. 6f, g). These findings demonstrate that *Nk*Chit2b- 1 disrupts multiple life-cycle stages of beet armyworm, including egg hatching, larval feeding behavior, and growth physiology, highlighting its potential as a biocontrol agent in agricultural pest management.

## Discussion

Chitinases represent a promising class of biocontrol agents with significant potential for agricultural pest and disease management (Karasuda et al. 2003; Veliz et al. 2017). However, current research on chitinases primarily focuses on species-specific resistance assays, while cross-kingdom efficacy in diverse host–pathogen systems remains poorly characterized. In this study, we purified a chitinase, *Nk*Chit2b- 1, from the digestive fluid of *Nepenthes* pitcher plants using a prokaryotic expression system (Fig. 1b). This enzyme exhibited high catalytic activity within the pH window (5.0–6.0) and temperature range (28–42 °C) that aligns with terrestrial plant growth conditions (Fig. 1c, d). Notably, the optimal pH range of *Nk*Chit2b- 1 corresponds to the extracellular pH fluctuations in plant cell (5.6–5.7 under basal conditions and pH 6.0 upon pathogen challenge) (Masachis et al. 2016), suggesting its functional relevance in plant immunity. Despite comparable enzymatic activity to rice chitinase *Os*Chit1 and *Arabidopsis* chitinase *At*HCHIB across tested parameters (4–50 °C; pH 3.0–8.0) (Figs. 1c, d, S1a, b), *Nepenthes*-derived *Nk*Chit2b- 1 demonstrates over tenfold higher prokaryotic expression yield (Fig. S1c). This remarkable biosynthetic efficiency confers superior cost-effectiveness for scalable production, positioning NkChit2b- 1 as a promising candidate for developing eco-friendly biological agents for plant protection. Subsequent physiological and pathogenicity assays confirmed that *Nk*Chit2b- 1 not only effectively inhibits the growth of various pathogenic fungi, including *S. sclerotiorum* (Fig. 2a, b), *B. cinerea* (Fig. 3a, b), and *M. oryzae* (Fig. 4a, b), but also influenced the behavior of BPH (Fig. 5) and beet armyworm (Fig. 6) in multiple ways. Our findings align with previous studies highlighting the role of plant chitinases in degrading chitin-rich structures of pathogens and pests (Karasuda et al. 2003; Veliz et al. 2017). Furthermore, exogenous application of *Nk*Chit2b- 1 conferred potent broad-spectrum disease and pest resistance across diverse plant species, including *Arabidopsis* (Figs. 2c, d, 3c, d, and 6b-e), tobacco (Fig. 3e, f), tomato (Fig. 2e, f), and rice (Figs. 4c, d and 5a). Notably, *Nk*Chit2b- 1 conferred protection against *B. cinerea* and *M. oryzae* in a dose-dependent manner, with enhanced efficacy observed at elevated protein concentrations (Figs. 3c, d, S2, 4c, d). Mechanistically, these findings establish *Nk*Chit2b- 1 as a multifunctional defense molecule capable of simultaneously targeting fungal pathogens and herbivorous pests. Collectively, its remarkable cross-kingdom activity, combined with environmental safety profiles, positions this chitinase as a promising candidate for sustainable crop protection, aligning with global demands for eco-friendly biopesticide.

As endogenous pathogenesis-related (PR) proteins, chitinases are massively secreted during plant immune responses to biotic stress, and their exogenous application poses no harm to plant tissues (Vaghela et al. 2022). Based on previous research (Gong et al. 2020), we can speculate that the chitin fragments generated by *Nk*Chit2b- 1 may be recognized by plant immune receptors, further reinforcing plant defense responses. In addition, compared to traditional chemical pesticides, *Nk*Chit2b- 1 exhibit advantages including lower toxicity, environmental friendliness, and reduced non-target organism harm, contributing to ecological balance and mitigating pesticide resistance. These attributes make *Nk*Chit2b- 1 particularly suitable for organic and sustainable agriculture.

While *Nk*Chit2b- 1 exhibited potent activity against tested pathogens and pests, it failed to significantly inhibit the growth of *F. oxysporum* (Supplementary Fig. S3), a soil-borne fungus causing wilt diseases in multiple crops. The differential chitinase susceptibility observed in fungal pathogens can be explained by the spatial heterogeneity of chitin accessibility within cell walls. Specifically, glucan-encapsulated chitin domains act as physical barriers that restrict enzymatic access (Cortés et al. 2019; Liu et al. 2020). To address this, future studies could engineer *Nk*Chit2b- 1 variants with enhanced substrate penetration or combine it with β-glucanases to synergistically degrade fungal walls. Similarly, given the vast diversity of chitinases in nature, combining multiple chitinases could yield synergistic effects. Tailored chitinase formulations targeting specific pathogens or pests may further enhance their efficacy. To translate these findings into field applications, challenges such as enzyme stability under fluctuating environmental conditions must be addressed. Encapsulation technologies or fusion proteins could prolong *Nk*Chit2b- 1 activity in planta. Furthermore, exploring synergistic combinations with other PR proteins or microbe-associated molecular patterns (MAMPs) (e.g., glucanase proteins or *At*pep1) may broaden its target spectrum and delay resistance evolution.

Taken together, *Nk*Chit2b- 1 represents a versatile biocontrol tool with dual antifungal and insecticidal properties. Its plant-compatible biochemical profile and eco-friendly mode of action position it as a promising candidate for sustainable agriculture.

## Supplementary Information

Supplementary Material 1.

## Data Availability

Data available on request from the authors.
