# Supplementary material for: Nepenthes chitinase NkChit2b- 1 confers broad-spectrum resistance to chitin-containing pathogens and insects in plants
Source: Adv Biotechnol (Singap). 2025 Apr 21;3(2):12. doi: 10.1007/s44307-025-00066-8 (PMC12011681; doi:10.1007/s44307-025-00066-8)
Supplement: Supplementary file 1 — Supplementary Material 1. [file 44307_2025_66_MOESM1_ESM.docx]

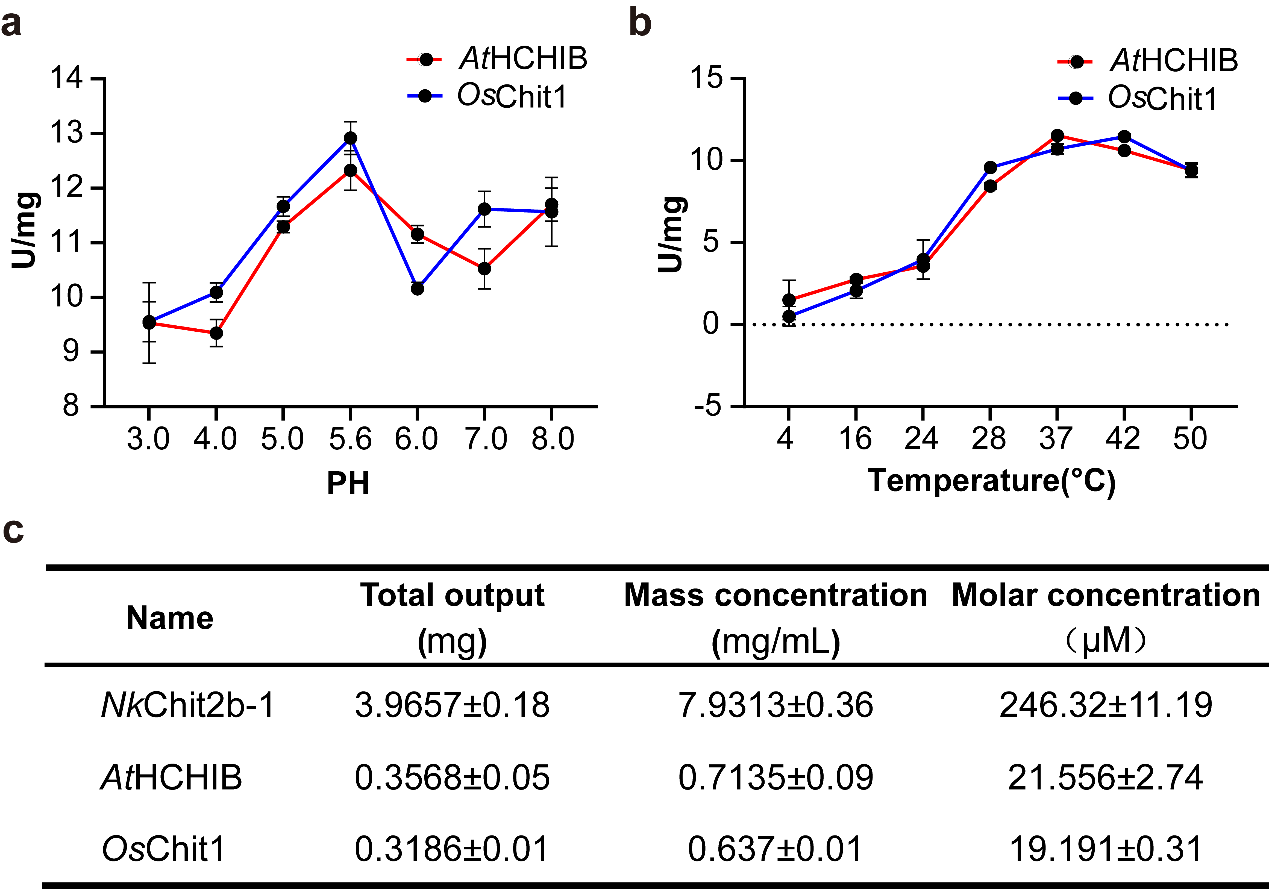


**Figure. S1 Enzymatic activity profiling of chitinase *At*HCHIB and *Os*Chit1**

**a** Effect of pH on the enzymatic activity of *At*HCHIB and *Os*Chit1. Activity was assayed at pH 3.0, 4.0, 5.0, 5.6, 6.0, 7.0, and 8.0. **b** Effect of temperature on the enzymatic activity of *At*HCHIB and *Os*Chit1. Activity was evaluated at 4°C, 16°C, 24°C, 28°C, 37°C, 42°C, and 50°C. Data are presented as mean ± SEM (n = 3). **c** Comparison of recombinant chitinase yields in prokaryotic expression systems. Data are presented as mean ± SEM (n = 2).


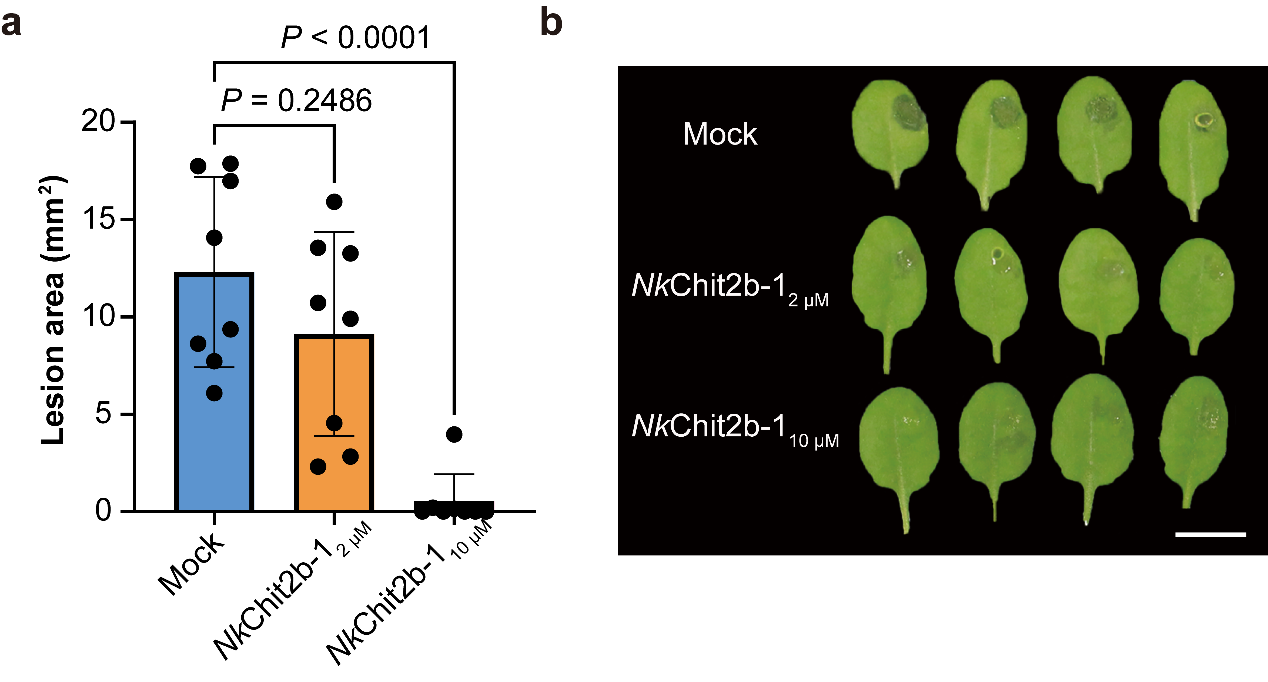


**Figure. S2** ***Nk*Chit2b-1 confers concentration-dependent resistance to*B. cinerea*in*Arabidopsis***

**a** Statistical analysis of disease lesion areas in *Arabidopsis*. Scale bar = 1 cm. Data are presented as mean ± SEM (n = 8). Data were processed using one-way ANOVA, with *P* indicating the significance of differences. **b** *Nk*Chit2b-1 confers concentration-dependent resistance to *B. cinerea* in *Arabidopsis*. Disease symptoms in *Arabidopsis* leaves were observed 24 h after inoculation. Scale bar = 1 cm.


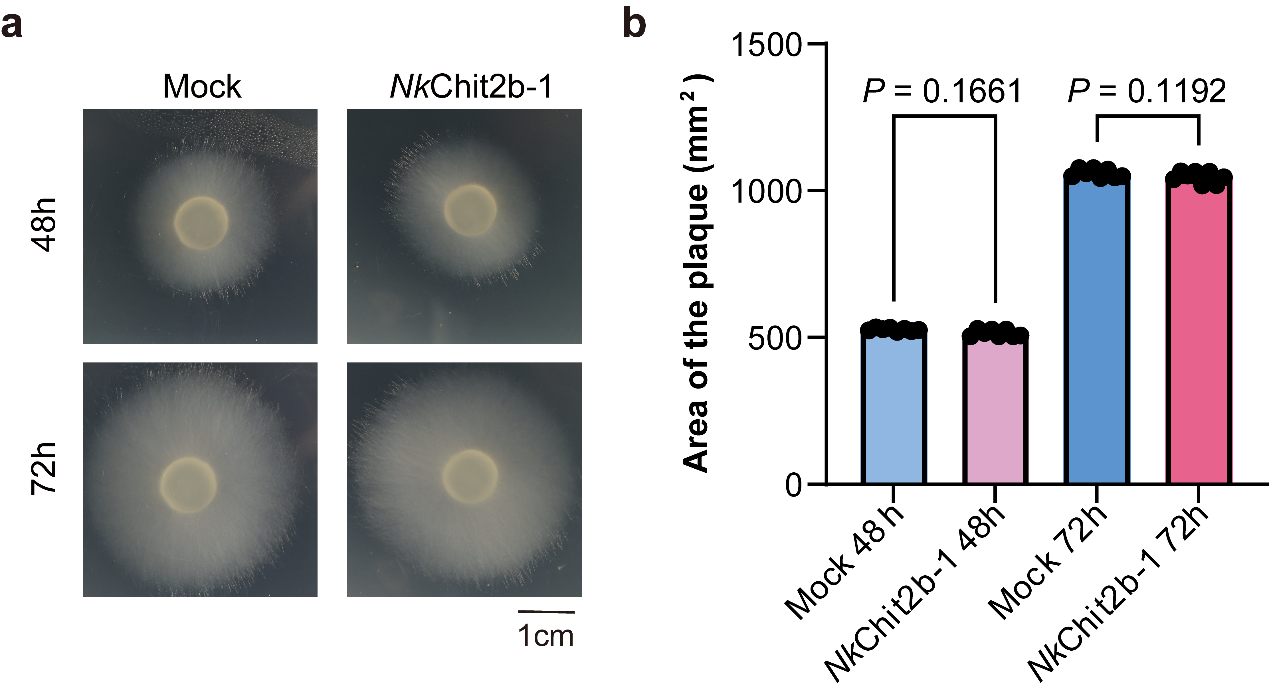


**Figure. S3 Limited effect of *Nk*Chit2b-1 on the mycelial growth of *F. oxysporum***

**a** *Nk*Chit2b-1 treatment on Fo5176 mycelial growth. Representative images at 48 h and 72 h post-inoculation. Scale bar = 1 cm. **b** Quantitative analysis of Fo5176 plaque area. Data are presented as mean ±SEM (n = 9). Data were processed using one-way ANOVA, with *P* indicating the significance of differences.
